# Non-Radiative Carrier Recombination Enhanced by Two-Level Process: A First-Principles Study

**DOI:** 10.1038/srep21712

**Published:** 2016-02-16

**Authors:** Ji-Hui Yang, Lin Shi, Lin-Wang Wang, Su-Huai Wei

**Affiliations:** 1 National Renewable Energy Laboratory, Golden, CO 80401, USA; 2 Suzhou Institute of Nano-Tech and Nano-Bionics, Chinese Academy of Sciences, Suzhou 215125, China; 3 Materials Sciences Division, Lawrence Berkeley National Laboratory, Berkeley, CA 94720, USA; 4 Beijing Computational Science Research Center, Beijing 100094, China

## Abstract

Non-radiative recombination plays an important role in the performance of optoelectronic semiconductor devices such as solar cells and light-emitting diodes. Most textbook examples assume that the recombination process occurs through a single defect level, where one electron and one hole are captured and recombined. Based on this simple picture, conventional wisdom is that only defect levels near the center of the bandgap can be effective recombination centers. Here, we present a new two-level recombination mechanism: first, one type of carrier is captured through a defect level forming a metastable state; then the local defect configuration rapidly changes to a stable state, where the other type of carrier is captured and recombined through another defect level. This novel mechanism is applied to the recombination center 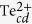 in CdTe. We show that this two-level process can significantly increase the recombination rate (by three orders of magnitude) in agreement with experiments. We expect that this two-level recombination process can exist in a wide range of semiconductors, so its effect should be carefully examined in characterizing optoelectronic materials.

Non-radiative carrier recombination, also known as Shockley-Read-Hall (SRH) recombination^1^,^2^,^3^,^4^, is a phenomenon that plays a key role in understanding semiconductor physics. In optoelectronic devices such as solar cells and light-emitting diodes (LEDs), non-radiative recombination can reduce device efficiencies by reducing the collection of photo-generated carriers and carrier lifetimes or suppressing luminescence^5^. In a non-radiative recombination process, one electron (hole) is first trapped by defects through defect transition energy levels with the help of phonons and then one hole (electron) is trapped, leading to carrier recombination, as shown in Fig. 1. In general, if the defect level is close to the conduction band minimum (CBM), electrons can be more easily trapped, but it will be very difficult to trap holes. Similarly, holes can be easily trapped and electron trapping is difficult when the defect level is close to the valance band maximum (VBM)^6^. Consequently, the recombination process is determined by the slower trapping rate of the electron or hole. So when the densities of electrons and holes in a material are similar under working conditions, such as in a CdTe solar cell, it is natural to assume that defects with defect levels close to the middle of the bandgap are the most important recombination centers; this is because these defects can trap both electrons and holes in similar rates. However, due to the lack of accurate numerical calculations of the recombination rate, few quantitative studies exist to verify this assumption. Furthermore, in device modeling, the non-radiative recombination rate is often estimated^7^,^8^, because direct experimental measurement of such processes is often difficult^9^,^10^,^11^. Recently, Shi *et al*.^6^,^12^ and Alkauskas *et al*.^13^ have developed *ab-initio* theories to calculate the non-radiative recombination rates by efficiently calculating the electron-phonon coupling in semiconductors. In their study, they assumed that the recombination process is determined only by the minority-carrier capture rates. However, in a more general situation, both electron and hole capture processes should be considered in the same footing, especially when the electron and hole concentrations are in similar orders, and the final recombination rate is determined by the slower capture process. This is because the carrier capture rate *U*_*c*_ is the product of defect densities *N*_*D*_, carrier densities *n* for electrons or *p* for holes, and carrier capture rate constant *B*, written as *U*_*pc*_ = *B*_*p*_*pN*_*D*_ for holes or *U*_*nc*_ = *B*_*n*_*nN*_*D*_ for electrons^4^. Moreover, previous calculations assume that the recombination occurs through a single defect level. However, in reality, a defect can have more than one defect configuration and defect energy level when it has multi-charged states. For example, in p-type CdTe, the Te on Cd antisite defect, Te_Cd_, is most stable in the 2+ state with a T_d_ symmetry. After it traps one electron and becomes a 1+ state, it can adopt either a metastable T_d_ symmetry structure inherited from the 2+ charged state or adopt a stable distorted C_3v_ ground-state structure due to the structural relaxation (see Fig. 2). Therefore, the electron trapping may occur by jumping from the T_d_ 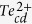 state either to a T_d_ 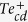 state with the defect transition energy level at point A or to a C_3v_ 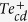 state with the defect transition energy level at point B. Similarly, the hole trapping can occur through the defect transition energy level at point B or point C. As a result, the validity of the assumption that the whole recombination process occurs through a single defect level is questionable. Besides, current *ab-initio* theories of calculating non-radiative recombination rate adopt a harmonic approximation, which requires that the trapping process preserves the original symmetry of the defect before and after carrier trapping. Consequently, the conventional wisdom that only those defects with deep levels can be effective recombination centers needs to be revisited and the detailed recombination process needs to be carefully considered.

In this paper, we show that a two-level non-radiative recombination process, among all possible phonon-assisted recombination processes, could generally exist in semiconductor systems. Using p-type CdTe as an example, we show that this mechanism could account for the non-radiative recombination through Te_Cd_ antisite defects. During the recombination, an electron is fast trapped through a level close to the CBM while a hole is fast trapped through a level close to the VBM. The two trapping processes are connected by a fast structural transformation. As a result, the whole recombination process is greatly accelerated, and unlike previously thought, defects with relatively shallow defect levels such as the Te_Cd_ (+/2+) level in CdTe could cause strong recombination. Our work also provides a general routine to systematically determine the recombination process in a system with all the parameters obtained entirely from first-principles calculations.

## Results

### Determination of potential recombination centers

As a first step, the non-radiative recombination center candidates should have significant densities in the system. To determine the defect densities, we considered all the intrinsic defects in CdTe. Because the formation energies of charged defects are functions of Fermi energies, we self-consistently solved the detailed balance equations under equilibrium growth conditions. Here, we used a typical value of 800 K as the growth temperature. After the sample is grown, it is cooled rapidly to the working temperature of 300 K. It is assumed that during the rapid cooling process, the total density of the defects is fixed and only different charge states of the same defects can redistribute accordingly. The detailed calculations can be found in ref. 14. For p-type CdTe grown under Cd-poor conditions, our calculations show that only four defects have significant densities at the working temperature: *V*_*cd*_, 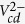, 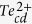, and 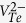, with densities of 1.03 × 10^15^/cm^3^, 1.66 × 10^14^/cm^3^, 2.15 × 10^13^/cm^3^ and 5.56 × 10^13^, respectively (see ref. 14), and the hole density is 1.77 × 10^14^/cm^3^ with the Fermi energy of 0.34 eV above the VBM.

**Figure 2:**
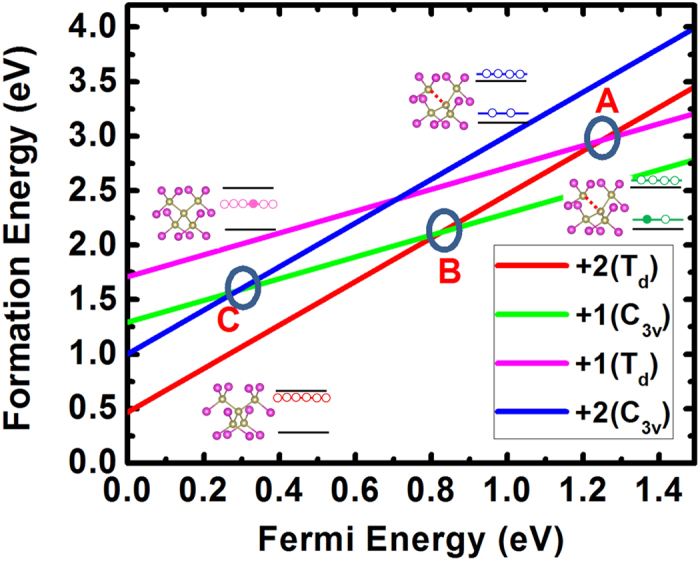
HSE06 calculated formation energies of Te on Cd antisite at +2 and +1 charge states in p-type CdTe as functions of Fermi energies (referenced to the VBM). The inset shows the atomic and electronic configurations for different states. Pink balls are Cd and light yellow balls are Te.

**Figure 3:**
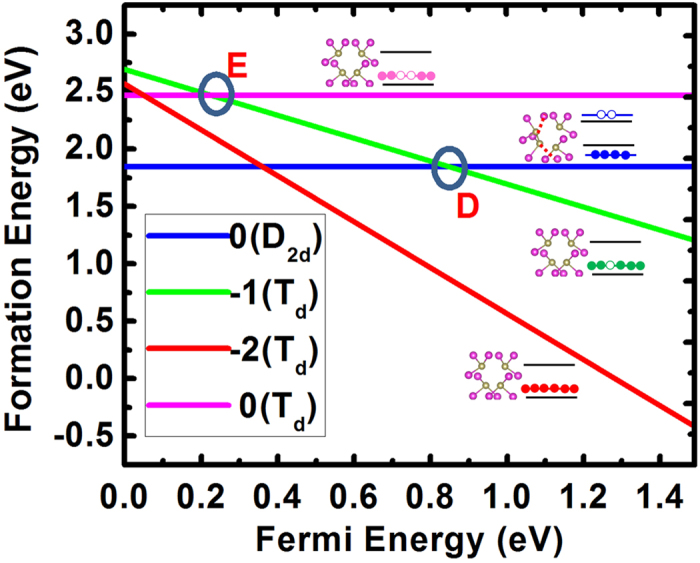
HSE06 calculated formation energies of Cd vacancy at different charge states and structural configurations as functions of Fermi energies (referenced to the VBM). The inset shows the atomic and electronic configurations for different states. Pink balls are Cd and light yellow balls are Te.

To be an effective recombination center, a defect should have a defect transition level close to the CBM when it traps an electron and have a defect level close to the VBM when it traps a hole. If only a single level is involved in both processes, this implies that the defect level should be close to the middle of the bandgap, as the SRH model suggests. (The probability of trapping more than one carrier is usually very small and such cases are not considered here.) In considering these, we find that the (+2/+1) transition energy level of 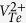 is above the CBM, and the (−2/−) transition energy level of 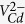 is below the VBM; therefore, both 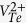 and 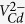 cannot be effective recombination centers and only 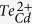 and *V*_*Cd*_ can be candidates as carrier recombination centers as shown in Figs 2 and 3. Because these two defects can only trap electrons, the first step of the whole recombination process should be the electron-trapping process. Only after electron trapping is hole trapping meaningful. Our following discussions are thus based on this basic procedure.

For 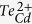, it is stable in the T_d_ symmetry with its three degenerated defect states unoccupied and close to the CBM, as shown in Fig. 2. The first step, as we discussed above, is the electron-trapping process. After 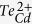 traps one electron and becomes 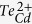, this 1+ state can exist in two different atomic structures. One is the original T_d_ symmetry structure with one electron occupying the three degenerated defect states. In this case, the electron trapping occurs through a (2+/+) level at point A in Fig. 2 with a value of 1.24 eV above the VBM, thus close to the CBM. The other is the C_3v_ structure, in which Te moves along the direction of one Te-Cd bond, splitting the three degenerated defect states into one state with one electron occupation and two fully unoccupied degenerated *e* states. Relative to the T_d_ symmetry structure, the energy gain due to this structure distortion is 0.42 eV; thus, the (2+/+) level is pushed down toward the VBM. In this case, the electron trapping could also occur through point B in Fig. 2, with a value of 0.82 eV above the VBM. Finally, the 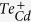 state will be stabilized at the C_3v_ structure, as we discuss below. After the electron trapping, the second step is hole trapping. When 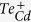 traps one hole and becomes 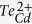, the 2+ state can also exist in the C_3v_ structure and T_d_ symmetry structure. As a result, the hole trapping can occur through defect levels at point C and point B with (+/2+) values of 0.29 eV and 0.82 eV above the VBM, respectively.

For neutral *V*_*Cd*_, the D_2d_ structural distortion splits the three degenerated states (each can occupy two electrons) in T_d_ symmetry into two degenerate occupied *e* states and one fully unoccupied *a* state with an energy gain of about 0.62 eV, as shown in Fig. 3. At the first step, neutral *V*_*Cd*_ with D_2d_ symmetry needs to trap one electron and becomes 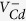. However, the total energy of the 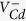 state with D_2d_ structure configuration is very high due to the high defect state caused by structural distortion, making the (0/−) level well above the CBM (not shown in Fig. 3). As a result, non-radiative electron trapping through this level is unlikely considering the energy-conservation requirement. In this case, we only consider the electron trapping by jumping from the D_2d_ *V*_*Cd*_ state to the T_d_ 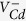 state through the defect transition energy level at point D in Fig. 3. Due to the high position of the *a* state, the (0/−) defect transition energy level from neutral D_2d_ *V*_*Cd*_ to T_d_ 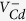 is relatively deep at 0.85 eV above the VBM of CdTe. After the electron trapping, 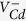 can then trap one hole and become neutral 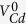, which can be either in a T_d_ symmetry state or a D_2d_ symmetry state. Consequently, the hole trapping can occur through defect levels at points E and D in Fig. 3 with (−/0) levels of 0.22 eV and 0.85 eV above the VBM, respectively.

From the above discussion, the non-radiative recombination center candidates can be systematically determined as 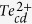 and neutral V_Cd_ and the recombination process can involve their possible defect levels at points A, B, and C in Fig. 2 and points D and E in Fig. 3. Considering the basic procedure that an electron is trapped first and then a hole can be trapped, we conclude that the dominant electron-trapping process can occur through defect levels at points A, B, and D, and the dominant hole-trapping process can occur through defect levels at points B, C, D, and E. Other processes are not important and thus ignored during the following discussions.

### Determination of photo-generated carrier densities

**Figure 4:**
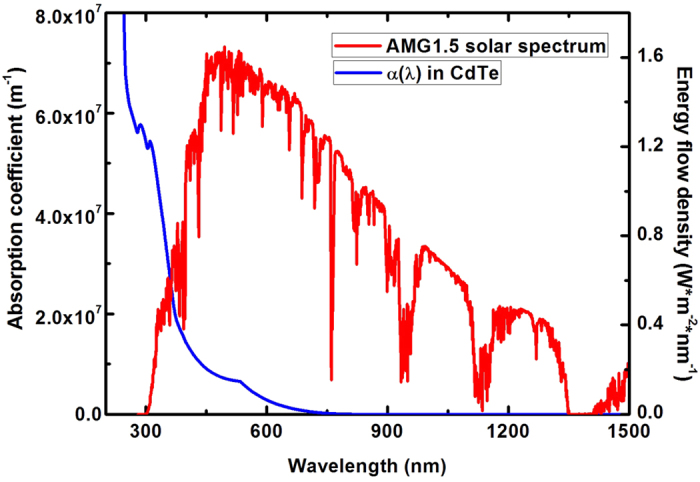
HSE06 calculated optical absorption coefficients of CdTe as functions of light wavelengths. The AM1.5G solar spectrum is also given.

A complete recombination process should involve both electron and hole trapping. As a result, both electron and hole trapping should, in principle, be considered and the slower process determines the whole recombination rate. This is true if hole density and electron density in a system are similar, as we discussed in the introduction. As a result, we should know the amount of holes and electrons in p-type CdTe under working conditions to see if we need to consider both the trapping processes of electrons and holes or if we just need to consider the minority-carrier trapping. To determine the amount of photo-generated carriers in CdTe, we have calculated the wavelength-dependent optical absorption coefficients *α*(*λ*) using the dielectric functions obtained from first-principles calculations, as shown in Fig. 4. We can then estimate the photo-generated electron density *n*_*G*_ and hole density *p*_*G*_ by:


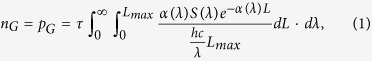
where *S*(*λ*) is the energy flow densities of solar emission, *τ* is the carrier lifetime in CdTe, *h* is Planck’s constant, *c* is light velocity, and *L*_*max*_ is the thickness of the CdTe absorber layer. Here, we use the standard AM1.5G solar spectral irradiance distribution for *S*(*λ*) and a typical value of 2 *μ*m for *L*_*max*_. Using the typical carrier lifetime in the order of 100 *ns* for bulk CdTe^15^,^16^, which is also confirmed by our following calculations, we estimate that the photo-generated carrier densities are 4 × 10^14^/cm^3^. Knowing that the intrinsic hole density in CdTe without sunlight is calculated to be 1.77 × 10^14^/cm^3^, the total carrier densities *p* and *n* under sunlight are not significantly different. Therefore, both electron and hole trapping should be considered, and the slower process will determine the final recombination rate.

### Calculations of carrier capture rate constants

Using the newly developed theoretical calculation methods for carrier capture rate through multi-phonon emissions (MPE) by Shi *et al*.^6^,^12^ and with static approximations^17^,^18^, we have calculated the carrier capture rate constants through the above-mentioned levels for holes and electrons. For each process, we used the phonon modes and electron-phonon coupling constants based on the initial states as an approximation within the harmonic phonon approximation. For example, for electron trapping through the defect level at point A, we obtained phonon modes and the electron-phonon coupling constants of these phonon modes from the T_d_ configuration because both the initial state and final state has T_d_ symmetry; for hole trapping through level C, the C_3v_ configuration is used to get phonon modes and the corresponding electron-phonon coupling constants because the initial 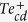 state has C_3v_ configuration.

First, we consider the electron trapping through defect levels at points A, B, and D. As shown in Table 1, the electron-trapping rate constants at T = 300 K through points A, B, and D are 2.50 × 10^−7^ *cm*^*3*^*/S*, 1.69 × 10^−10^ *cm*^*3*^*/s*, and 3.54 × 10^−10^ *cm*^*3*^*/s*, respectively, calculated using the harmonic approximation. The reason for the large electron-trapping rate constant through the defect level at point A is that this level is much closer to the CBM, so it can easily capture electrons. The relatively small electron-trapping rate constants through defect levels at points B and D mainly result from the defect levels being 0.67 eV and 0.64 eV from the CBM, respectively, which are much deeper. Considering that the defect density of 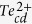 is 2.15 × 10^13^ /*cm*^3^ and the density of V_Cd_ is 1.03 × 10^15^/*cm*^3^, the electron SRH lifetimes, calculated as τ = (*B*_*n*_*N*_*D*_)^−1^, are 186 ns, 2.75 × 10^5^ ns, and 2.74 × 10^3^ ns, for electron trapping through defect levels at points A, B, and D, respectively. Compared to the carrier lifetime due to radiative recombination in CdTe, which ranges from 780 ns to 8.8 *μ*s^16^, only the non-radiative electron trapping through the defect level at point A is significant.

**Figure 5:**
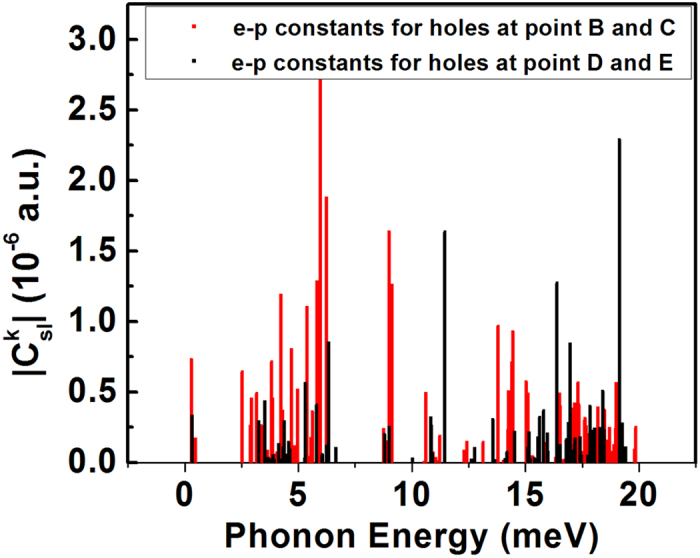
Calculated electron-phonon coupling constants between the VBM states and defect states at Γ point for holes as functions of phonon energies at points B and C inFig. 2 and points D and E in Fig. 3, respectively.

Next, we consider the hole trapping through defect levels at points B, C, D, and E. The hole-trapping rate constants at T = 300 K through defect levels at points B, C, D, and E, are 1.67 × 10^−7^ *cm*^*3*^*/s*, 2.46 × 10^−6^ *cm*^3^/*s*, 1.55 × 10^−11^ *cm*^3^/*s*, and 3.95 × 10^−8^ *cm*^3^/*s*, respectively. The hole-trapping rate constant through the defect level at point C is very large because this point is much closer to the VBM. More importantly, the C_3v_ distortion leads to strong electron-phonon coupling between the single occupied defect state (derived from conduction band) and the VBM states. The reason is that the distortion causes considerable mixing of *s* character on the substitutional Te site, thus allowing strong electron transition from this level to the VBM states dominated by *p* character. Moreover, the distortion is localized on this substitutional Te site, which allows strong phonon excitations after hole trapping. Similarly, the hole-trapping rate constant through the defect level at point B is also relatively large, but due to its relatively deep level, it is one order smaller than that at point C. The hole-capture constants through defect levels at points D and E are much smaller compared to those at points B and C, although their levels are similar, i.e., points B and D are very close and points C and E are very close. This is because at points D and E, the coupling (the dipole matrix element) between the three degenerated defect states with mostly *p* character (derived from the valance band) shared by four neighboring Te and the VBM is weak, which results in smaller electron-phonon coupling constants compared to those at points B and C. As clearly seen in Fig. 5, hole trapping through defect levels at points B and C have larger electron-phonon coupling constants than that through defect levels at points D and E in general, especially at regions of low phonon energy. Because the hole trappings are often accompanied by low-energy phonon modes, the larger hole-capture rate constant through defect levels at points B and C are expected due to its larger *e-p* coupling constants at regions of low phonon energy. Again, we can estimate the hole SRH lifetimes through defect levels at points B, C, D, and E to be 279 ns, 18.9 ns, 6.26 × 10^4^ ns, and 24.6 ns, respectively.

## Discussion

From the above discussion and considering that the whole recombination process can only start from electron trapping due to 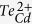 and neutral *V*_*Cd*_, we can exclude neutral *V*_*Cd*_ as dominant recombination centers, because electron trapping through the defect level at point D is very slow. However, for 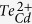, if electrons and holes are recombined through only one level (such as point B) as has usually been assumed, it is also impossible for 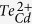 to cause strong recombination in CdTe because electron trapping through the defect level at point B is also very slow. As a result, we need to explore the possibility that the recombination occurs through more than one level, i.e., electron trapping occurs through the defect level at point A while hole trapping through the defect level at point B or C. We notice that the energy barrier from the T_d_ 1+ state to the C_3v_ 1+ state is actually (close to) zero, which means that the time needed for the structural transition is very short compared to the time to trap one hole. Then we can expect that the defect, which needs a relatively longer time to trap one hole, will soon change to another configuration after trapping one electron and before hole trapping. This is also why we did not consider the hole trapping at point A in the previous sections. One might wonder whether trapping one electron through the defect level at point A then relaxing to the C_3v_ 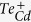 state is the same process as directly trapping one electron through the defect level at point B. The easy relaxation from the T_d_ 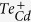 state to C_3v_ 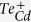 state after accepting one electron can be considered as strong electron-phonon coupling, which can enhance the electron-trapping rate through the defect level at point B. Nevertheless, our direct calculation indicates that, under harmonic approximation of the phonon mode, the direct electron trapping through the defect level at point B is slow. This could be a breakdown of the phonon harmonic approximation (for trapping through point B) that has been used in our formalism (whereas the trapping through point A can be described by the harmonic approximation). But it could also be that the electronic configuration of the C_3v_ 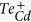 state can be considered as a different electronic configuration compared to the case of the T_d_ 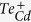 state (then the transition at defect levels A and B are clearly distinct). In either case, it means that a fast harmonic oscillator-like (e.g., as described by our multi-phonon quantum formula, or the classical Marcus theory) direct transition through the defect level at point B is impossible. For the transition at point A, after the defect traps one electron and becomes 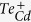, the phonon degree of freedom should quickly relax to the T_d_ ground state because it has the same symmetry as before the transition and it is a relatively much smaller displacement (in other words, the T_d_ 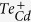 state is a local basin in the high-dimensional energy manifold of the phonon degree of freedom after the charge transition, which only has one degree of freedom leading to the C_3v_ 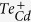 state). After that, it could then relax to the C_3v_ 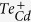 state. Thus, it will be more descriptive and meaningful to describe the process as first trapping one electron through the defect level at point A with T_d_ symmetry then quickly relaxing to the ground state with C_3v_ symmetry. The same can be said for trapping a hole through the defect level at point C with C_3v_ symmetry, then relaxing to the ground state of 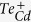 with T_d_ symmetry (Fig. 2). The hole trapping through the defect level at point C is easy because the 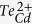 state has the same C_3v_ symmetry as the 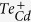 state. The difference is that the energy barrier from the 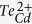 state with C_3v_ symmetry (blue line in Fig. 2) to the 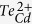 state with T_d_ symmetry (red line in Fig. 2) is now 0.23 eV according to our nudged elastic band^19^ calculations based on HSE06 functional^20^. Using the transition state theory^21^, we can estimate the time it takes for the 2+ state to transform from the C_3v_ structure to the T_d_ structure, which is given by 
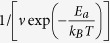
, where *E*_*a*_ is the energy barrier and *ν* is the transition rate. Using *E*_*a*_  = 0.23 eV and the calculated *v* = 1.02 THz, the time needed to complete the transition at T = 300 K is about 6.81 ns, which is less than the time costs for the hole and electron trapping. This is reasonable and consistent with the fact that the dominant state of 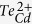 has T_d_ symmetry in p-type CdTe; otherwise, there could be accumulations of 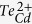 state with C_3v_ symmetry during the recombination process if the barrier is very large.

Finally, we describe the whole recombination process mediated by 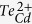 through two levels in p-type CdTe as follows: First, the electron trapping, which is also the key step, occurs through that defect level at point A with a large capture rate constant and changes the T_d_ 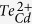 state to be T_d_ 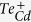 state. Second, the T_d_ 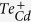 state changes to the C_3v_ 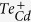 state with nearly zero barriers due to structural relaxation or phonon vibration. Third, the hole trapping occurs through the defect level at point C with a large capture rate constant and makes the C_3v_ 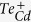 state change to the C_3v_ 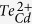 state. Fourth, the C_3v_ 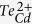 state changes to the T_d_ 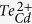 state by overcoming an energy barrier of 0.23 eV with the help of phonon vibrations, finishing the whole recombination loop. The entire process is determined mainly by the slowest step. According to our calculation, the slowest step in the 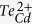 recombination process is the first step, which has an estimated carrier lifetime of 186 ns. We notice that our result is in good agreement with a recent experimental measurement of intrinsic p-type CdTe grown by molecular beam epitaxy^16^, which has a hole density of 3 × 10^14^/*cm*^3^ and a bulk SRH lifetime of 360 ns. As a result, among all the possible recombination mechanisms if any, our proposed recombination mechanism by two levels can provide reasonably good explanations and a detailed understanding of the entire process.

## Conclusions

We have systematically studied the non-radiative recombination process in intrinsic CdTe using first-principles methods. Contrary to the conventional wisdom (SRH model), which assumes that the electrons and holes recombine through a single defect level, we find that in some cases a two-level recombination process could be the dominant process. That is, electron capture occurs through one level near the CBM at a large rate and hole capture occurs through the other level near the VBM, also at a large rate. These two trapping processes are connected by a fast structural transformation of the defect, which has different stable and metastable configurations at different charge states. Our results of carrier recombination in CdTe are in good agreement with experiments. Because it is quite common for defects in semiconductors to have different stable and metastable configurations at different charge states, we expect our proposed two-level recombination mechanism should play an important role in other semiconductor systems. Therefore, our work provides new insights and guidelines for the future study of defect physics in semiconductors.

## Methods

### First-principles calculations of defect properties

Our first-principles calculations were performed using density-functional theory (DFT)^22^,^23^ as implemented in the VASP code^24^,^25^. The electron and core interactions are included using the frozen-core projected augmented wave (PAW) approach^26^. To correct the bandgap error, we used the Heyd-Scuseria-Ernzerhof (HSE06) hybrid functional^20^. For all the HSE06 supercell calculations, all the atoms are fully relaxed until the forces on every atom are less than at least 0.05 eV/Å. To determine the defect formation energies and defect transition energy levels, we calculated the total energy *E*(*α, q*) for a CdTe supercell containing the relaxed defect *α* in its charge state *q*. We also calculated the total energy *E*(*CdTe*) for the same supercell in the absence of the defect, as well as the total energies of elemental solids or gases at their stable phases. The defect formation energy Δ*H*_*f*_ (*α, q*) as a function of the electron Fermi energy *E*_*F*_ and the atomic chemical potentials *μ*_*i*_ is given by^27^:


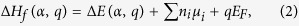
where Δ*E*(*α, q*) = *E*(*α, q*) − *E*(*CdTe*) + *n*_*i*_*E*(*i*) + *qE*_*VBM*_, *E*_*F*_ is referenced to the VBM of bulk CdTe, and *μ*_*i*_ is the chemical potential of constituent *i* referenced to elemental solid or gas with energy *E*(*i*). The *n*’*s* are the numbers of atoms taken out of the supercell to form the defects, and *q* is the number of electrons transferred from the supercell to the Fermi reservoirs in forming the defect cell. The defect transition energy level *ε*_*α*_(*q*/*q*′) is the Fermi energy *E*_*F*_ in Eq. (2) at which the formation energy Δ*H*_*f*_ (*α, q*) of defect *α* at charge *q* is equal to that of the same defect at another charge state *q*′, i.e.,


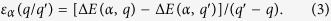
To get fast convergence on total energy and transition energy levels and good descriptions on the symmetry of the defect state, we used a mixed scheme^27^ to determine *ε*_*α*_(*q*/*q*′). In this scheme, for an acceptor (*q* < 0), the ionization energy level with respect to the VBM is given by


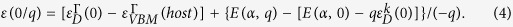
For donor levels (*q* > 0), the ionization energy referenced to the CBM is given by


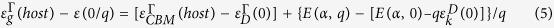
where 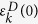 and 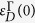 are the defect levels at the special *k* points (averaged) and at the Γ point, respectively; 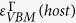 and 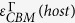 are the VBM and CBM energy, respectively, of the pure CdTe supercell at the Γ point. The first term on the right-hand side of Eq. (4) or (5) gives the single-electron defect level at the Γ point. The second term determines the *U* energy parameter (including both the Coulomb contribution and atomic relaxation contribution) of the charged defect calculated at the special *k* points, which is the extra cost of energy after moving (−*q*) charge to the neutral defect level with 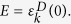 Once we know the defect transition energy levels, the formation energy of a charged defect is then given by


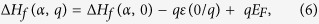
where Δ*H*_*f*_ (*α*,0) is the formation energy of the charge-neutral defect and *E*_*F*_ is the Fermi level with respect to the VBM. To evaluate the effect of image interactions of charged defects and supercell size, we carefully check the transition energy levels in 256-atom supercells and 512-atom supercells and make sure the defect transition energy levels are well converged. Although the defect levels can affect the calculated recombination trapping rate constants, our proposed two-level recombination mechanism should not be affected. For the calculations of optical transition energy levels, we constrained the symmetry to be same before and after the carrier is trapped, which is required by the harmonic approximation.

### Electron-phonon coupling calculation methods

The electron-phonon coupling parts are calculated following the method described in refs 6 and 12. based on static approximations^17^,^18^. The nonradiative decay probability between the initial electronic state *i* and the final electronic state *j* is given by the conventional Fermi “golden rule” expression:


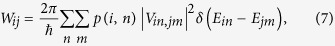
where *V*_*in*,*jm*_ are the off-diagonal matrix elements of the total Hamiltonian defined as 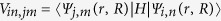, and *p*(*i, n*) is the probability that the system is in the initial state Ψ_*i*,*n*_(*r, R*), so that ∑_*n*_ *p*(*i, n*) = 1. Provided that the vibrational equilibrium rate considerably exceeds the nonradiative decay rate, *p*(*i, n*) can be described by Boltzmann distribution:


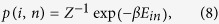
where 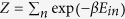 is the partition function and *β* = (*k*_*B*_*T*)^−1^. Under static approximations^17^,^18^, *V*_*in*,*jm*_ can be written as:


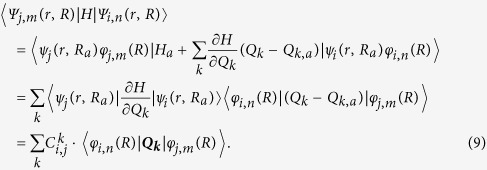
Here, *R*_*a*_ is the relaxed atomic position of state *i* or *j* and *Q*_*k*,*a*_ is normal phonon modes of electronic state *i* or *j*. By definition, ***Q***_***k***_ ≡ *Q*_*k*_ − *Q*_*k*,*a*_, is atomic vibration for the phonon state *k*, which can be calculated for electronic state *i* and *j* as:


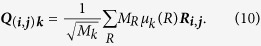
Here, *R*_*a*_ is the *k*-th phonon mode vector, and *M*_*R*_ is the nuclear mass for atom at *R*. Under harmonic approximation, the phonon modes for electronic state *i* and *j* are the same and we have:


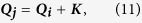
where 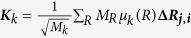. Here **Δ*****R***_***j***,***i***_ = ***R***_***j***_(0) − ***R***_***i***_(0) is the relaxed atomic position difference of the system at the electronic states *i* and *j*. Then the electron phonon coupling constant between electronic states *i* and *j* and phonon mode *k* is:


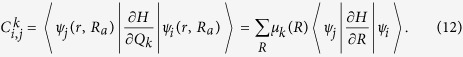
And the phonon modes satisfy:


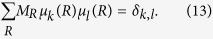
We calculated all the zone-centered phonon modes using density-functional perturbation theory using PBE functionals^28^ as implemented in VASP. For the term 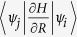, we used our recently proposed variational method^6^,^12^. For a local/semilocal exchange-correlation functional, the Kohn-Sham Hamiltonian is written as:


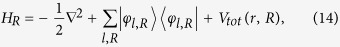
where *φ*_*l,R*_ is the nonlocal potential projector for atom *R* and angular momentum *l*. Thus:


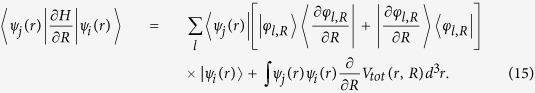


The first term is the same term as in Hellman-Feynman force evaluation, which can be calculated readily. For the second term, we have used 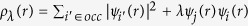 and we prove that 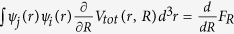, with *F*_*R*_ being ab initio atomic force on atom *R* calculated from the Hellman-Feynman formula while keeping *ρ*_*λ*_(*r*) fixed during SCF iterations. This part is calculated using PEtot code.

After we obtain the nonradiative decay probability *W*_*ij*_, the capture rate constant is *B* = *W*_*ij*_ ⋅ *V*, where *V* is the volume of the supercell. Our previous results are 4.30 × 10^−8^ and 1.46 × 10^−7^ *cm*^*3*^*/s* for GaP:Zn_Ga_-O_P_ in a 64-atom supercell and GaN:Zn_Ga_-V_N_ in a 72-atom supercell^12^, which are in good agreement with the experimental results, which are 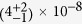 and 3.0 × 10^−7^ *cm*^*3*^*/s*^29^,^30^, respectively. We estimate the accuracy of this work is similar to our previous work using a 64-atom CdTe supercell.

### Energy barrier and transition rate calculation methods

We calculated the related energy barriers using the Heyd-Scuseria-Ernzerhof (HSE06) hybrid functionals^20^ (α = 0.25) and nudged elastic band (NEB) method^19^ as implemented in VASP. Five images are used. After the local minimum and transition state are obtained, the transition rate *ν* for a defect or particle to overcome its barrier is defined as^21^:


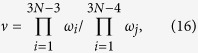
where *ω*_*i*_ are 3*N* − 3 positive phonon frequencies at the local minimum configuration and *ω*_*j*_ are 3*N* − 4 positive phonon frequencies at the saddle point. At this step, the zone-centered phonon frequencies at the minimum sites and saddle points are calculated within the PBE^28^ framework by making finite displacements (0.015 Å) and we fixed the atoms, which are more than 5 Å away from the defect centers, as we did in ref. 31. and our results there agree well with available experimental measurements. The transition state, according to its definition, has only one imaginary phonon frequency. As a result, we calculated the phonon frequencies of all the considered states in this work. If one imaginary phonon frequency is found for some state, the energy barrier is assigned to zero. For example, the C_3v_ 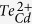 state has one imaginary phonon frequency; as a result, the barrier from C_3v_ 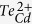 state to T_d_ 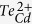 state is zero.

## Additional Information

**How to cite this article**: Yang, J.-H. *et al*. Non-Radiative Carrier Recombination Enhanced by Two-Level Process: A First-Principles Study. *Sci. Rep.* **6**, 21712; doi: 10.1038/srep21712 (2016).

## Figures and Tables

**Figure 1:**
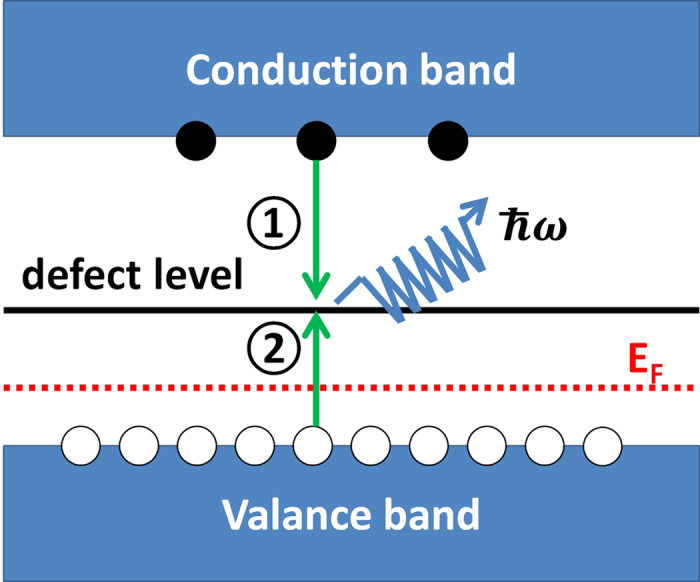
Diagram to show the recombination process in a p-type material. First, the defect level captures one electron from the CBM, releasing phonons; then, the defect level captures one hole from the VBM, releasing phonons and completing the recombination. This diagram is drawn by J.-H. Y.

**Table 1:** Calculated electron capture rate constants *B*_*n*_ and hole capture rate constants *B*_*p*_ through defects levels at points A, B, C, D, and E in Figs 2 and 3.

Level	*B*_*n*_(*cm*^3^ *s*^−1^)	*B*_*p*_(*cm*^3^ *s*^−1^)
A(Te_Cd_)	2.50 × 10^−7^	–
B(Te_Cd_)	1.69 × 10^−10^	1.67 × 10^−7^
C(Te_Cd_)	–	2.46 × 10^−6^
D(V_Cd_)	3.54 × 10^−10^	1.55 × 10^−11^
E(V_Cd_)	–	3.95 × 10^−8^
